# Prediction of Immune Infiltration Diagnostic Gene Biomarkers in Kawasaki Disease

**DOI:** 10.1155/2022/8739498

**Published:** 2022-06-17

**Authors:** Hongjun Ba, Yao Wang, Lili Zhang, Huishen Wang, Zhan-Peng Huang, Youzhen Qin

**Affiliations:** ^1^Department of Paediatric Cardiology, Heart Centre, First Affiliated Hospital of Sun Yat-Sen University, 58# Zhongshan Road 2, Guangzhou 510080, China; ^2^Key Laboratory on Assisted Circulation, Ministry of Health, 58# Zhongshan Road 2, Guangzhou 510080, China; ^3^Guangzhou Medical University Cancer Hospital, Guangzhou 510095, China

## Abstract

Kawasaki disease (KD) is characterized by disorder of immune response with unknown etiology. Immune cells may be closely related to the onset of KD. The focus of this research was to evaluate the significance of the infiltration of immune cells for this disease and find possible diagnostic biomarkers for KD. The Gene Expression Omnibus database was utilized to retrieve two freely accessible gene expression patterns (GSE68004 and GSE18606 datasets) from human KD and control specimens. 114 KD, as well as 46 control specimens, were searched for obtaining differentially expressed genes (DEGs). Candidate biological markers were determined utilizing the support vector machine recursive feature elimination and the least absolute shrinkage and selection operator regression model analysis. To assess discriminating capacity, the area under the receiver operating characteristic curve (AUC) was computed. The GSE73461 dataset was utilized to observe the biomarkers' expression levels and diagnostic significance in KD (78 KD patients and 55 controls). CIBERSORT was employed to assess the composition profiles of the 22 subtypes of immune cell fraction in KD on the basis of combined cohorts. 37 genes were discovered. The DEGs identified were predominantly involved in arteriosclerotic cardiovascular disease, atherosclerosis, autoimmune disease of the urogenital tract, and bacterial infectious disease. Gene sets related to complement and coagulation cascades, Toll-like receptor signaling pathway, Fc gamma R-mediated phagocytosis, NOD-like receptor signaling pathway, and regulation of actin cytoskeleton underwent differential activation in KD as opposed to the controls. KD diagnostic biomarkers, including the alkaline phosphatase (ALPL), endoplasmic reticulum degradation-enhancing alpha-mannosidase-like protein 2 (EDEM2), and histone cluster 2 (HIST2H2BE), were discovered (AUC = 1.000) and verified utilizing the GSE73461 dataset (AUC = 1.000). Analyses of immune cell infiltration demonstrated that ALPL, EDEM2, and HIST2H2BE were linked to CD4 memory resting T cells, monocytes, M0 macrophages, CD8 T cells, neutrophils, and memory CD4 T cells. ALPL, EDEM2, and HIST2H2BE could be utilized as KD diagnostic indicators, and they can also deliver useful information for future research on the disease's incidence and molecular processes.

## 1. Introduction

Kawasaki disease (KD) is an acute-onset systemic febrile disease and vasculitis occurring in childhood that may result in coronary artery lesions (CALs) [1]. This illness mostly influences children under the age of five and has emerged as the major contributor to acquired heart disease in children across numerous industrialized nations [2]. The origin of KD is still a mystery, notwithstanding the widespread investigation throughout the last five decades. Numerous research reports have recently shown that the pathogenic mechanisms of KD are linked to infection, genetic vulnerability, and immunological responses, and several current studies are focusing on its therapy on the basis of multiple pathogenic hypotheses. According to some studies, KD results from pathogenic bacteria infection, which causes abnormal immune system activation and the corresponding cascade production of inflammatory mediators [3]. Research has confirmed that monocyte performs an instrumental function in vasculitis [4, 5]. Contemporary research has also revealed the significant function of regulatory T cells as a crucial indicator for both the susceptibility and severity of KD [6]. Some genes, such as ITPKC, CASP3, and FCGR2A, have been shown to be intimately linked to the regulation of immune function in KD [7, 8]. Immunogenetics performs an integral function in the pathogenic mechanism of KD, as evidenced by these findings.

Over the past few years, microarray technologies have been used in combination with integrated bioinformatics analysis to find new genes linked to a variety of disorders that could serve as diagnostic as well as prognostic biomarkers [9–13]. For instance, CASP5 has been found to be a potential biomarker for KD [14]. In addition, the PTEN/PI3K/VEGF signaling pathway was found to be involved in the vascular damage in a rabbit KD model [15].

Furthermore, research has revealed that infiltration of immune cells is becoming particularly crucial in the onset and progression of numerous diseases [10, 16–18]. Nevertheless, few research reports have utilized CIBERSORT to examine immune cell infiltration in KD as well as prospective diagnostic indicators for the disease.

We employed the Gene Expression Omnibus (GEO) database to acquire KD microarray datasets for this investigation. To screen and find diagnostic indicators for KD, machine learning techniques were applied. Based on gene expression levels, CIBERSORT was utilized to determine the percentages of immune cells in specimens of KD and normal specimens in this investigation. Additionally, we assessed the link between the discovered biological markers and infiltrating immune cells in order to lay the groundwork for future investigation.

## 2. Materials and Methods

### 2.1. Microarray Data

The GSE68004 [19] and GSE18606 [20] datasets were acquired as a series of matrix files from http://www.ncbi.nlm.nih.gov/geo/. The GSE68004 dataset contained 76 KD and 37 controls derived from circulatory endothelial cells, while the GSE18606 dataset contained 38 KD and 9 controls derived from peripheral blood. Predicated on the probe annotations data, each dataset probe was converted into gene symbols. The average of the probe was determined as the gene's final expression level when over one probe corresponded to the same gene symbol. Since they share an identical domain and are important for merging data derived from disparate datasets, these 2 datasets were integrated into a metadata cohort for subsequent integrative study. In addition, the batch effect was eliminated utilizing the “SVA” package of R software's combat function [21]. Furthermore, the Illumina HumanHT-12 V4.0 expression beadchip was utilized to validate the GSE73461 dataset [22], which was derived from peripheral circulation and contained 78 KD and 55 control specimens.

### 2.2. Screening for DEGs and Processing of Data

The 2 datasets were integrated into a metadata cohort, and preprocessing and elimination of batch effects were accomplished utilizing the SVA program's combat function. To perform array background correction, differential expression, and normalization analyses between 114 KD and 46 control specimens, we utilized the R program limma (http://www.bioconductor.org/). DEGs were defined as specimens having a ∣log fold change (FC) | ≥1 and an adjusted false discovery rate (FDR) *p* < 0.05.

### 2.3. Analysis of Functional Enrichment

The DOSE and “clusterProfiler” packages in R were utilized to undertake disease ontology (DO) enrichment analysis on DEGs [23, 24]. The highly essential functional keywords between the KD and control subgroups were determined utilizing gene set enrichment analysis (GSEA). DEGs are introduced to the gene ontology (GO) independently in bioinformatics website (https://maayanlab.cloud/enrichr/) and KEGG analysis [25]. The standard gene set was “c2.cp.kegg.v7.4.symbols.gmt” from the Molecular Signatures Database (MSigDB). A gene set was considered highly enriched in the case when *p* value < 0.05 and the FDR < 0.025.

### 2.4. Scanning for Potential Diagnostic Biomarkers

Two machine learning algorithms were employed to determine illness status and detect relevant prognostic factors. The least absolute shrinkage and selection operator (LASSO) is a regularization-based regression analysis approach that improves predictive performance. The R software's “glmnet” function was utilized to carry out the LASSO regression analysis to find the genes that were substantially linked to the differentiation of KD and normal specimens. The support vector machine (SVM) is a supervised machine learning approach that is commonly utilized for regression and categorization. The RFE method was utilized to identify the optimum genes derived from the metadata cohort to minimize overfitting [26]. Support vector machine recursive feature elimination (SVM-RFE) was utilized to determine the optimal parameters in order to discover the gene sets exhibiting the greatest discriminative performance. Afterward, the expression profiles of putative genes were verified in the GSE73461 dataset, which comprised overlapped genes between both algorithms.

### 2.5. Featured Biomarkers' Diagnostic Significance in KD

We created a ROC curve utilizing mRNA expression values of 114 KD and 46 control specimens to examine the prognostic value of the identified biomarkers. The diagnostic significance in differentiating KD from control specimens was determined by computing the area under the ROC curve (AUC) value, which was then verified in the GSE73461 dataset.

### 2.6. Identification of Immune Cell Subsets

A bioinformatics technique named CIBERSORT (https://cibersortx.stanford.edu/) was employed to probe into the infiltration status of immune cells utilizing gene expression patterns in KD to measure the proportional numbers of infiltrating immune cells. A standard set of 22 kinds of immune cell subsets (IM22) having 1,000 permutations was utilized to assess the predicted abundance level of immune cells [27]. The R software's “corrplot” was utilized to undertake correlation analysis and representation of 22 different kinds of infiltrating immune cells. To display the differences in immune cell infiltration status between the KD and control specimens, violin plots were created with the help of the R package “vioplot.”

### 2.7. Analysis of Association between Immune Cell Infiltrates and Discovered Genes

The R program's Spearman's rank correlation analysis was adopted to examine the link between the gene markers that were identified and the level of immune cell infiltration. The identified relationships were shown utilizing the “ggplot2” program's chart approach.

### 2.8. Statistical Analysis

We utilized the R (version 3.6.3) to execute all analyses of statistical data. Regarding continuous data, group comparisons were made utilizing either Student's *t*-test for normally distributed data or the Mann-Whitney *U*-test for data having a nonnormal distribution. The R software's “glmnet” program was utilized for conducting LASSO regression analysis, while the “e1071” package was utilized for performing the SVM algorithm. ROC curve analysis was utilized to establish the diagnosis performance of the identified diagnostic indicators in the study. Spearman's correlation was utilized to examine the link between gene marker expression and infiltrating immune cells. All statistical analyses were two-sided with *p* < 0.05 serving as the determinant for statistical significance.

## 3. Results

### 3.1. Determination of DEGs in KD

**Figure 1:**
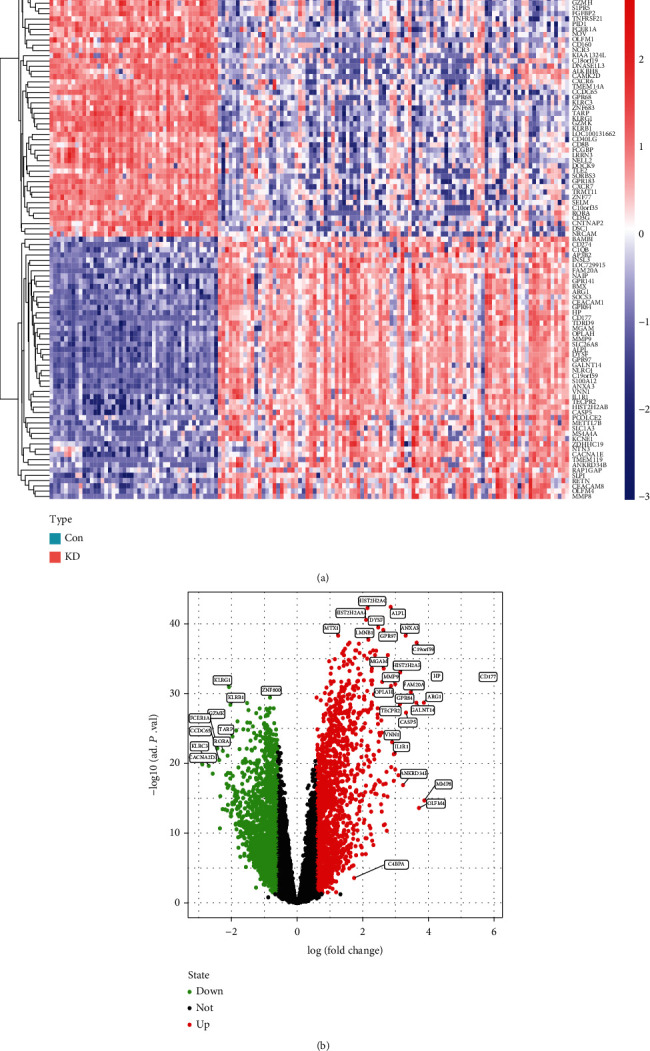
Differentially expressed genes between Kawasaki disease and normal samples. (a) Heatmap of top 100 up and top 100 down DEGs (Con was normal control group, and Treat was Kawasaki disease group). (b) Volcano plot of DEGs.

In this work, data from 114 KD and 46 control specimens were examined from 2 GEO datasets (GSE68004 and GSE18606 retrospectively). Upon eliminating batch effects, the metadata DEGs were examined with the limma program. A heatmap of the top 100 upregulated and top 100 downregulated DEGs is shown, and relative consistency was observed within groups (Figure 1(a)). In total, there were 37 DEGs retrieved: the expression of 27 genes was greatly upmodulated, whereas the expression of 10 genes was dramatically downmodulated (Figure 1(b)).

### 3.2. Functional Correlation Analysis

**Figure 2:**
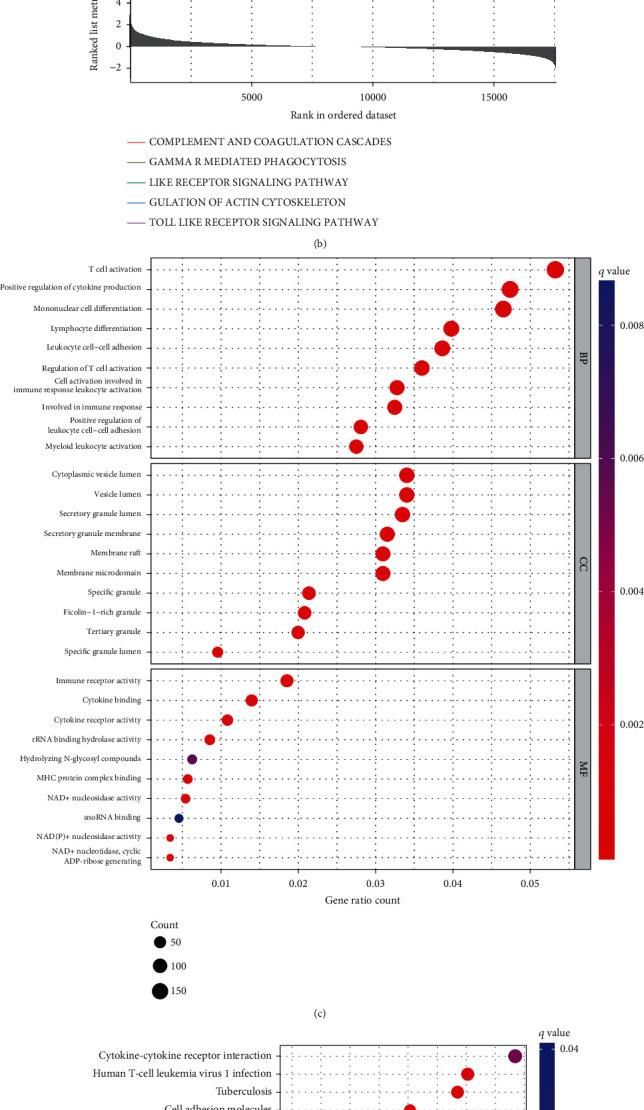
Functional enrichment analyses to identify potential biological processes via disease ontology and gene set enrichment analysis. (a) Disease ontology enrichment analysis of differentially expressed genes between KD and normal samples. (b) Enrichment analyses via gene set enrichment analysis. (c) Bubble plot of DEGs in KEGG. (d) GO of DEGs.

To examine the involvement of DEGs, DO pathway enrichment studies were undertaken. The findings revealed that DEG-enriched disorders were mostly linked to atherosclerosis, urogenital tract autoimmune disease, arteriosclerotic heart disease, and bacterial infectious illnesses (Figure 2(a)). The findings recorded from GSEA illustrated that the enriched pathways predominantly involved complement and coagulation cascades, Fc gamma R-mediated phagocytosis, Toll-like receptor signaling pathway, NOD-like receptor signaling pathway, and regulation of actin cytoskeleton (Figure 2(b)). GO and KEGG analyses indicated that these DEGs were closely related to T cell activation and cytokine production (Figures 2(c) and 2(d)). These data clearly show that immune responsiveness is critical in KD progression.

### 3.3. Diagnostic Biomarker Detection and Verification

**Figure 3:**
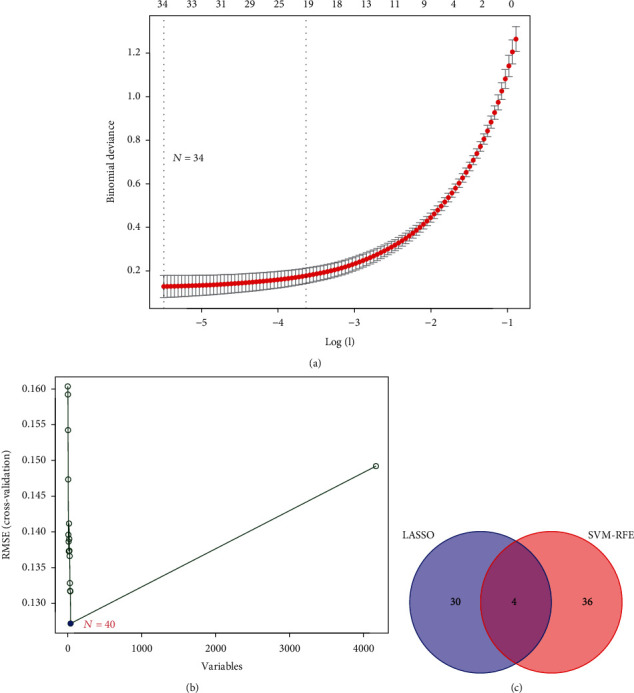
Screening process of diagnostic biomarker candidates for Kawasaki disease diagnosis. (a) Tuning feature selection in the least absolute shrinkage and selection operator model. (b) A plot of biomarkers selection via support vector machine-recursive feature elimination (SVM-RFE) algorithm. (c) Venn diagram demonstrating four diagnostic markers shared by the least absolute shrinkage and selection operator and SVM-RFE algorithms.

**Figure 4:**
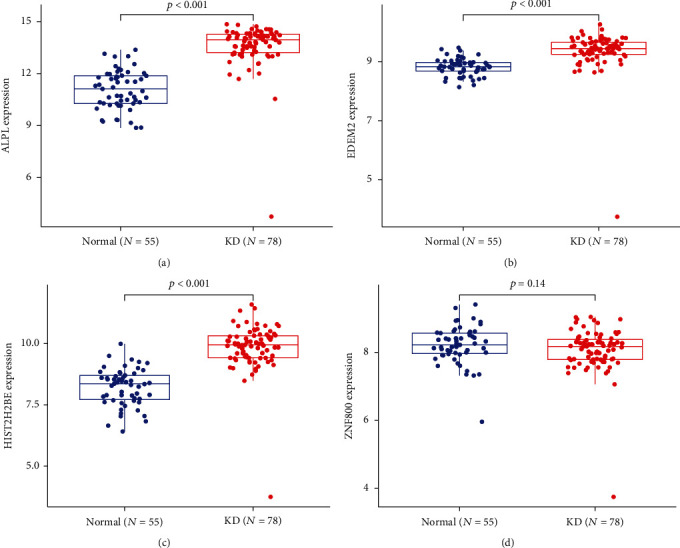
Validation of the expression of diagnostic biomarkers in the GSE73461 dataset. (a) ALPL, (b) EDEM2, (c) HIST2H2BE, and (d) ZNF800.

Possible biomarkers were screened utilizing two separate methods. Through the use of the LASSO regression technique, we narrowed down the DEGs, culminating in 34 factors as diagnostic markers for KD (Figure 3(a)). As illustrated in Figure 3(b), a subset of 40 DEG features was identified utilizing the SVM-RFE technique. In the end, the 4 overlapping characteristics between these 2 algorithms (ALPL, EDEM2, HIST2H2BE, and ZNF800) were chosen (Figure 3(c)). The GSE73461 dataset was also utilized to check the levels of expression of the four characteristics, resulting in highly accurate and robust results. The expression levels of ALPL, EDEM2, and HIST2H2BE in KD tissue were significantly greater as opposed to the controls (all *p* < 0.05, Figures 4(a)–4(c)). Nevertheless, when it came to ZNF800 expression, there was no remarkable difference across the 2 groups (Figure 4(d)). As a result, the 3 discovered genes were utilized to create a diagnosis model in the metadata cohort with the aid of a logistic regression approach.

### 3.4. Feature Biomarkers' Diagnostic Efficacy in KD

**Figure 5:**
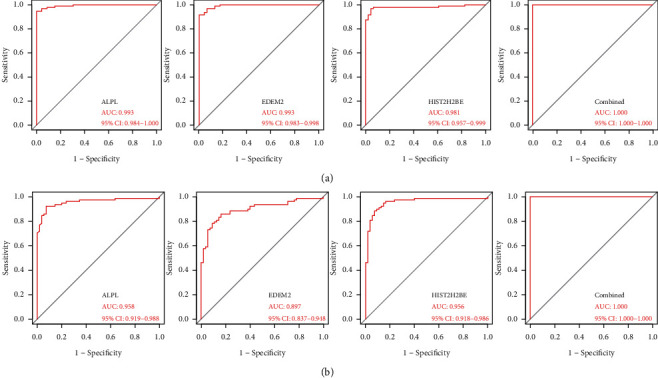
The receiver operating characteristic (ROC) curve of the diagnostic effectiveness of the three diagnostic markers. (a) ROC curve of ALPL, EDEM2, and HIST2H2BE after fitting to one variable in the metadata cohort. (b) ROC curve of ALPL, EDEM2, and HIST2H2BE after fitting to one variable in the GSE73461 dataset.

The diagnosing capacity of the 3 markers in distinguishing KD from control specimens exhibited a positive diagnostic significance, as illustrated in Figure 5(a), with AUC values of 0.993 in ALPL (95% CI 0.984–1.000), 0.993 in EDEM2 (95% CI 0.983–0.998), and 0.981 in HIST2H2BE (95% CI 0.957–0.999). In the metadata cohort, the diagnostic capacity expressed as AUC was 1.000 (95% CI 1.000–1.000) once the 3 genes were integrated into one variable. Additionally, a strong discriminating capacity was observed as illustrated in the GSE73461 dataset with AUC values of 0.958 in ALPL (95% CI 0.919–0.988), 0.897 in EDEM2 (95% CI 0.837–0.948), and 0.956 in HIST2H2BE (95% CI 0.918–0.986). Notably, the cumulative diagnostic performance of the 3 markers expressed as AUC was 1.000 (95% CI 1.000–1.000; Figure 5(b)), showing that the feature biological markers exhibited a strong diagnosing capacity.

### 3.5. Immune Cell Infiltration

**Figure 6:**
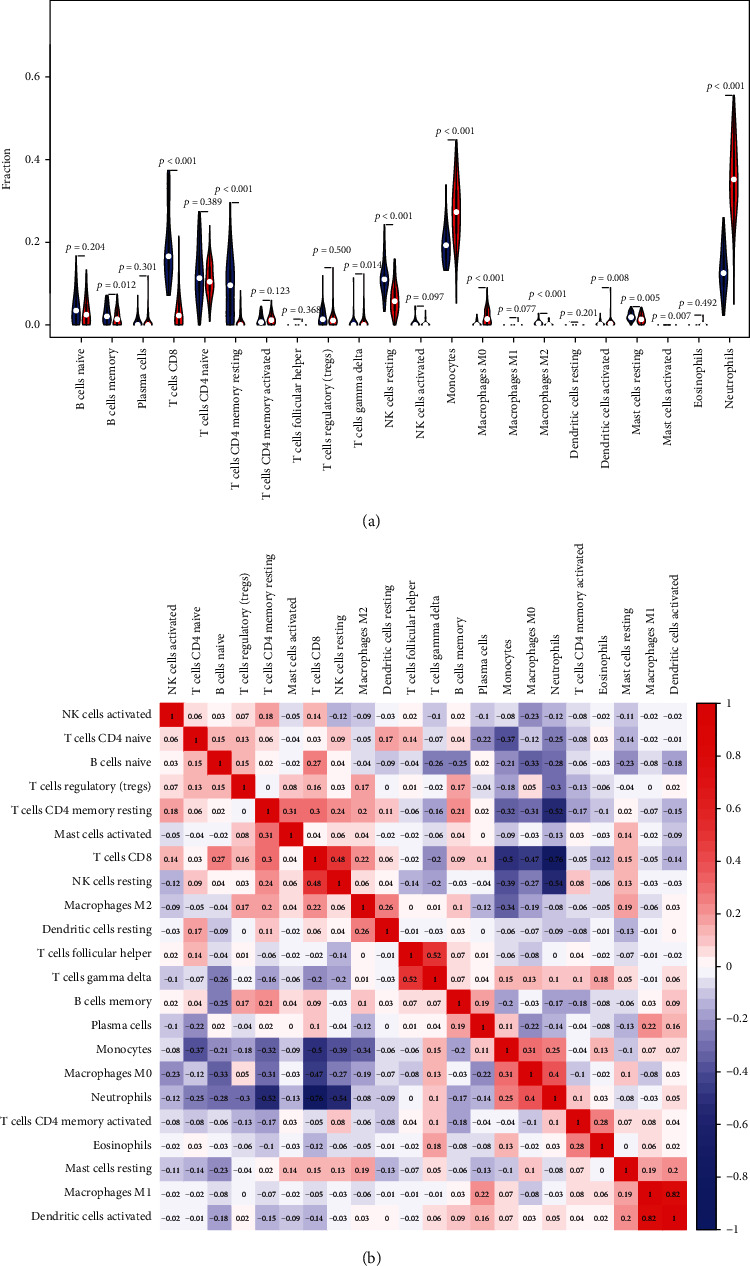
Distribution and visualization of immune cell infiltration. (a) Comparison of 22 immune cell subtypes between Kawasaki disease tissues and normal tissues. Blue and red colors represent normal and Kawasaki disease samples, respectively. (b) Correlation matrix of all 22 immune cell subtype compositions. Both horizontal and vertical axes demonstrate immune cell subtypes. Immune cell subtype compositions (higher, lower, and same correlation levels are displayed in red, blue, and white, respectively).

We began by examining the composition of the immune cell in KD relative to normal control tissues. As opposed to normal tissues, the percentages of CD8 T cells (*p* < 0.001), CD4 memory resting T cells (*p* < 0.001), resting NK cells (*p* < 0.001), and M2 macrophages (*p* < 0.001) in KD were considerably reduced. Additionally, the percentages of activated dendritic cells (*p* = 0.008), macrophages M0 (*p* < 0.001), monocytes (*p* < 0.001), gamma delta T cells (*p* = 0.014), and neutrophils (*p* < 0.001) in KD were substantially elevated as opposed to the normal samples (Figure 6(a)).

A correlation was computed between 22 different immune cell types (Figure 6(b)). CD4 memory resting T cells had a remarkable positive link to activated mast cells (*r* = 0.31, *p* = 0.004) but considerably inversely linked to neutrophils (*r* = −0.52, *p* < 0.001), M0 macrophages (*r* = −0.31, *p* < 0.001), and monocytes (*r* = −0.32, *p* < 0.001).

Follicular helper T cells were found to have a strong positive link to gamma delta T cells (*r* = 0.52, *p* = 0.04) and activated NK cells (*r* = 0.02, *p* = 0.01), but with a negative correlation with M0 macrophages (*r* = −0.08, *p* = 0.009).

Gamma delta T cells exhibited a strong positive link to follicular helper T cells (*r* = 0.52, *p* = 0.046) and a substantial negative link to naive B cells (*r* = −0.26, *p* < 0.001), CD4 memory resting T cells (*r* = −0.16, *p* < 0.001), and naive B cells (*r* = −0.26, *p* < 0.001).

Monocytes were shown to have a strong positive link to M0 macrophages (*r* = 0.31, *p* < 0.001) and neutrophils (*r* = 0.25, *p* < 0.001) and exhibited a strong negative link to CD8 T cells (*r* = −0.5, *p* < 0.001), resting NK cells (*r* = −0.39, *p* < 0.001), and CD4 naive T cells (*r* = −0.37, *p* < 0.001).

M1 macrophages exhibited a considerably positive correlation with plasma cells (*r* = 0.22, *p* = 0.001). Resting mast cells demonstrated a substantial positive relationship with M2 macrophages (*r* = 0.19, *p* = 0.016). Activated mast cells exhibited a substantial positive relation to memory resting CD4 T cells (*r* = 0.31, *p* = 0.004), and resting mast cells (*r* = 0.14, *p* = 0.04).

Neutrophils were found to have a considerable positive link to M0 macrophages (*r* = 0.4, *p* < 0.001) and monocytes (*r* = 0.25, *p* < 0.001) but had a considerable negative link to CD8 T cells (*r* = −0.76, *p* < 0.001), resting NK cells (*r* = −0.54, *p* < 0.001), and CD4 memory resting T cells (*r* = −0.52, *p* < 0.001).

### 3.6. Association between Immune Cell Infiltration and the Three Biomarkers

**Figure 7:**
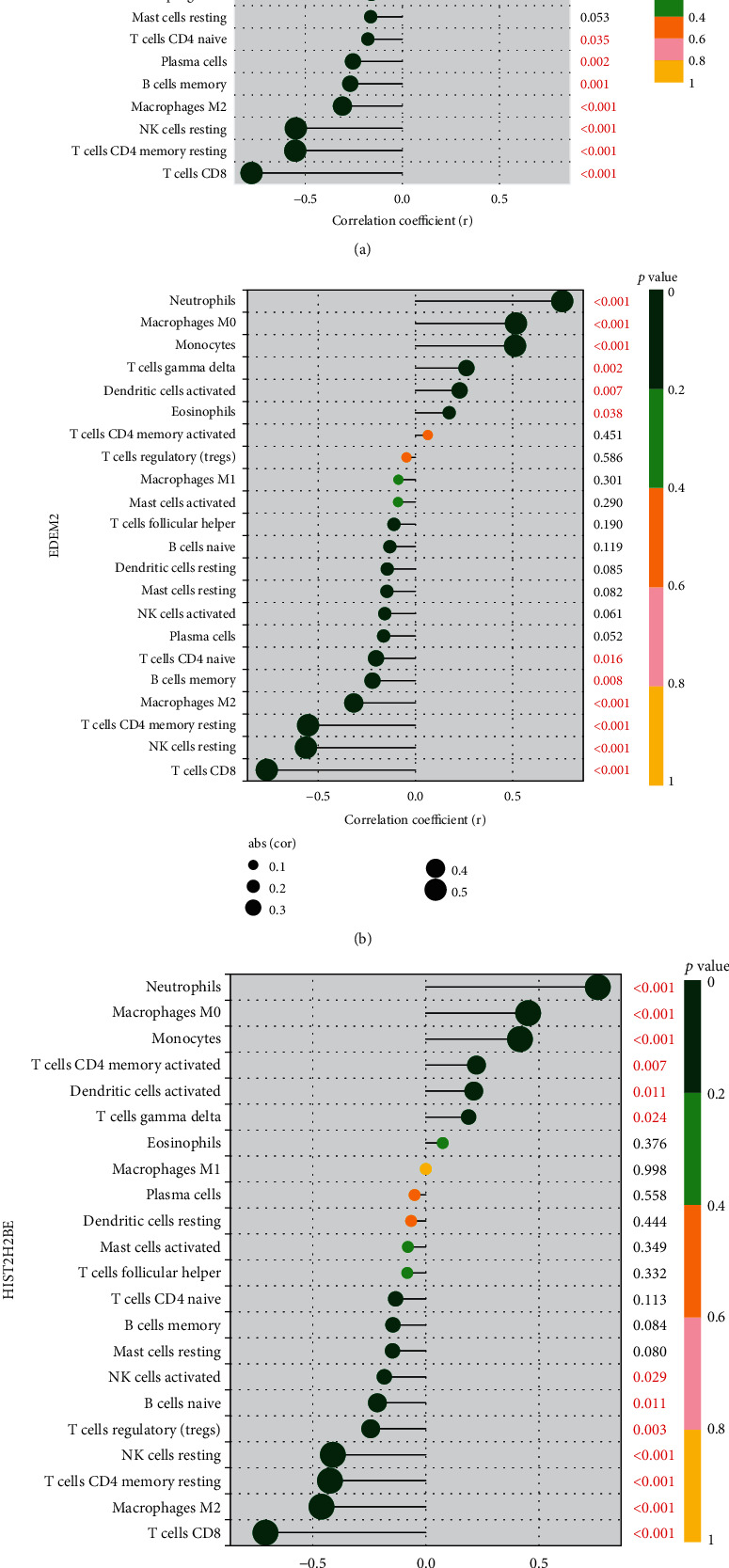
Correlation between ALPL (a), EDEM2 (b), HIST2H2BE (c), and infiltrating immune cells in Kawasaki disease.

As depicted in Figure 7(a), ALPL exhibited a strong positive link to neutrophils (*r* = 0.8, *p* < 0.001), M0 macrophages (*r* = 0.6, *p* < 0.001), monocytes (*r* = 0.52, *p* < 0.001), and gamma delta T cells (*r* = 0.22, *p* = 0.008) and negatively linked to CD8 T cells (*r* = −0.78, *p* < 0.001) and CD4 memory resting T cells (*r* = −0.55, *p* < 0.001). EDEM2 exhibited a positive link to neutrophils (*r* = 0.76, *p* < 0.001), M0 macrophages (*r* = 0.52, *p* < 0.001), and monocytes (*r* = 0.51, *p* < 0.001) and negatively linked to CD8 T cells (*r* = −0.76, *p* < 0.001), resting NK cells (*r* = −0.56, *p* < 0.001), and CD4 memory resting T cells (*r* = −0.55, *p* < 0.001) (Figure 7(b)).

HIST2H2BE has a positive link to neutrophils (*r* = 0.76, *p* < 0.001), M0 macrophages (*r* = 0.45, *p* < 0.001), monocytes (*r* = 0.42, *p* < 0.001), and CD4 memory activated T cells (*r* = 0.22, *p* = 0.0073) and a negative link to CD8 T cells (*r* = −0.71, *p* < 0.001), M2 macrophages (*r* = −0.46, *p* < 0.001), resting NK cells (*r* = −0.41, *p* = 0.0029), and CD4 memory resting T cells (*r* = −0.42, *p* < 0.001; Figure 7(c)).

## 4. Discussion

Despite the fact that remarkable advancement has recently been attained in the investigation and treatment of KD, numerous important subjects remain unanswered, including what causes the illness, how the CAL develops, and how IVIG therapy functions. The most significant clinical hallmark of acute KD is an explicit immunological response, which has aided in elucidating the disease's origin and progression process [28, 29]. Several immunological research, on the other hand, yielded contradictory results. Although IVIG can effectively prevent coronary artery disease in most children, there is still an elevated risk of CAL in some patients experiencing nonresponse from IVIG. Therefore, exploring the immunogenetic mechanism of Kawasaki disease is still the focus and difficulty of current research. Infiltration of immune cells has lately been proven as a significant factor in the onset and progression of KD. As a result, scientists are actively looking for new diagnostic biological markers and investigating the components of KD immune cell infiltrates, which might have a significant influence on KD patients' clinical results. MicroRNAs and mRNAs have recently been identified as prospective markers in cardiovascular diseases and particularly in KD [30].

Only few research have examined the abnormally expressed gene markers linked to infiltration level of immune cells in KD and normal specimens. As a consequence, we aimed to screen for possible KD diagnostic indicators and examine the function of infiltrating immune cells in the disease.

We utilized the GEO datasets to create two cohorts and executed a comprehensive analysis on them. In total, 37 DEGs were discovered, with 27 of them being upmodulated and 10 of them being downmodulated. The findings recorded from enrichment analyses illustrated that DEG-enriched illnesses were predominantly linked to autoimmune disease of the urogenital tract, arteriosclerotic cardiovascular disease, and bacterial infectious illness. As illustrated by the findings from the GSEA, the enriched pathways predominantly involved immune response and inflammatory pathways, including Fc gamma R-mediated phagocytosis, Toll-like receptor signaling pathway, and NOD-like receptor signaling pathway. These results are congruent with the prior findings that leukocyte-mediated inflammation performs a function in the etiology of KD [31]. In fact, KD is considered to be an immunomodulatory disorder induced by infection [32]. In the course of the acute phase of KD, a high proportion of inflammatory reactions were generated. Toll-like receptor pathways are implicated in the immunologic etiology of KD [33]. Through the IL-1R8 pathway, IL-37b reduces endothelial cell death and inflammatory responses in KD patients [34]. This data supports our findings, suggesting that the immune reaction is crucial in KD and validates the accuracy of the current study's findings. The immune response works primarily through effector immune cells.

To provide a safe and efficient therapy, careful control of diverse kinds of immune cells is required. As a result, bioinformatics analysis will help to identify new biomarkers of KD that are correlated with the degree of immune cell infiltration. Three diagnostic biomarkers were discovered utilizing 2 machine learning methods. The main function of the ALPL gene is to regulate tissue mineralization and also perform an integral function in cardiovascular remodeling [35, 36]. ALPL has the potential to be a new predictor of cardiovascular disease-related morbidity and mortality [37, 38]. Some studies have suggested that the mineralization metabolism regulated by ALPL gene expression is closely related to systemic inflammation. ALPL may play a crucial part in KD, based on the information presented above.

EDEM2, an important member of the endoplasmic reticulum (ER) degradation-enhancing *α*-mannosidase family, engages in fine-tuning the ER stress response, which influences ER survival and homeostasis [39]. ER stress response is considered a novel regulator of innate immunity. A link has been found between ER stress and a variety of human illnesses, including inflammatory diseases, infectious illnesses, cystic fibrosis, diabetes, and cancer.

Immunologic signaling pathways, including STATs, JNK, and NF*κ*B, are components of the interaction network of ER stress responsiveness. When oligomeric IRE1 is activated, it works by binding to the scaffold protein TRAF2, which is a key constituent of TNF-receptor signaling [40]. Histone variant HIST2H2BE is an important aging regulating protein, which is closely related to gliomas [41]. Currently, no studies have been conducted on its role in immune inflammation, and subsequent investigation is required.

CIBERSOTR was utilized to analyze the types of immune cell infiltrates in KD and normal specimens. As a consequence, a number of immune cell subgroups have been shown to engage in key biological functions in KD. An elevated infiltration level of monocytes, M0 macrophages, and neutrophils and reduced infiltration levels of CD8 T cells, resting NK cells, and CD4 resting memory T cells were shown to have a correlation with the incidence and progression of KD. Moreover, after completing an analysis of the link between ALPL, EDEM2, HIST2H2BE, and immune cells, they were all shown to have a substantial link to T cell gamma delta, M0 macrophages, monocytes, T cell CD8, neutrophils, and resting NK cells.

Inflammatory and immunological circulating cells, including lymphocytes, platelets, and neutrophils, have mostly been demonstrated to play a role in the advancement of heart disease [42, 43]. According to research, monocytes are the primary source of proinflammatory cytokines. Effector memory CD8+ T cells, as well as plasma cells, are also implicated in KD, with oligoclonal growth of T cell receptors (TCRs) and B cell receptors (BCRs) following IVIG treatment [44].

There are also research findings that B cell, T helper cell, and naive CD8+ T cell in KD children were reduced, while natural killer T (NKT) cells and immune-related T cells were increased [45]. In addition, SELL+CD14+CD16- monocytes were found to be expanded in KD [46].

The previously reported data, along with our current outcomes, have revealed that various kinds of infiltrating immune cells perform critical functions in KD and should be the subject of extensive research in the future. Although our study found that immune cell infiltration is closely related to the pathogenesis of Kawasaki disease, it is not clear what role immune cell infiltration plays in coronary artery injury, and further research is needed.

The study's limitations must be recognized. At first, because the research was retrospective, key clinical data was not accessible. Secondly, the small number of patients within the GSE18606 validation cohort ought to be recognized as a limitation. Additionally, biomarker patterns in blood and immune cell profiles were extracted from the two datasets, and their replicability ought to be subsequently tested. Lastly, bioinformatics analysis was used to deduce the activities of 3 biomarkers and infiltration status of immune cells in KD, and future investigation with bigger specimen sizes is necessary to corroborate our findings.

In conclusions, ALPL, EDEM2, and HIST2H2BE were linked to CD4 memory resting T cells, monocytes, M0 macrophages, CD8 T cells, neutrophils, and memory CD4 T cells. ALPL, EDEM2, and HIST2H2BE could be utilized as KD diagnostic indicators, and they can also deliver useful information for future research on the disease's incidence and molecular processes.

## Data Availability

This research evaluated public databases. All of the source data applied in this research came from the GEO data platform, which is open to the public (https://www.ncbi.nlm.nih.gov/geo/;Accessionnumbers: GSE68004, GSE18606, and GSE73461).

## Abbreviations

KD: Kawasaki disease
GEO: Gene Expression Omnibus
DEGs: Differentially expressed genes
ALPL: The alkaline phosphatase
EDEM2: Endoplasmic reticulum degradation-enhancing alpha-mannosidase-like protein 2
HIST2H2BE: Histone cluster 2, H2be
SVM-RFE: Support vector machine recursive feature elimination
AUC: Area under the receiver operating characteristic curve
CALs: Coronary artery lesions
FDR: False discovery rate
DO: Disease ontology
GO: Gene Ontology
GSEA: Gene set enrichment analysis
MSigDB: Molecular signatures database
LASSO: Least absolute shrinkage and selection operator.
